# Effects of mind-body exercises for osteoporosis in older adults: Protocol for systematic review and Bayesian network meta-analysis of randomized controlled trials

**DOI:** 10.1097/MD.0000000000019426

**Published:** 2020-03-13

**Authors:** Yonghui Zhang, Zhijie Wang, Min Lu, Qichao Wang, Haijiao Wang

**Affiliations:** a Department of Joint Surgery, Luohe Central Hospital, Luohe; b Shanxi Province Hospital of Traditional Chinese Medicine, Taiyuan; c Guangzhou University of Chinese Medicine, Guangzhou; d Orthopedics Department, the First Affiliated Hospital of Hunan University of Chinese Medicine, Hunan, China.

**Keywords:** mind-body exercise, network meta-analysis, older adults, osteoporosis

## Abstract

**background:**

Osteoporosis is an important cause of bone fractures and even a cause of threaten to the lives of elderly people. Mind-body exercises are beneficial interventions for improving flexibility, controlling body balance, and reducing pain. To assess the effect of mind-body exercise on osteoporosis in aging people, we will perform this systematic review.

**Methods:**

Randomized controlled trials (RCTs) which carried out about mind-body exercise for osteoporosis will be included. Web of Science, PubMed, Science Direct, Medline, Cochrane Library, China National Knowledge Infrastructure, and Wanfang will be searched from inception to January 2020. The outcomes will include bone mineral density (BMD), bone mineral content (BMC), body balance, function of lower extremity, pain, fearing level, and quality of life. Trial reporting quality will be assessed by 2 reviewers independently and Review Manager 5.3 software will be used for meta-analysis. Trial registration is under PROSPERO (CRD42020165385).

**Results:**

Based on the current evidence, the potential rank of the efficacy and safety of mind-body exercises for BMD, BMC, body balance, function of lower extremity, pain, fearing level and quality of life will be assessed, and a prioritization regimen will be summarized.

**Conclusions:**

Evidence from this systematic review could be useful for patients, clinical practitioners, and guideline-makers to select an optimum proposal of mild-body exercises for older adults with osteoporosis.

## 1 Introduction

### 1.1 Description of the condition

Up to 2017, there are almost 962 million aging people in the whole world according to World Population Prospects, and will increase to 2.1 billion in 2050.^[1]^ The morbidity of some disorders likes osteoporosis is growing with the growing age. Osteoporosis is a skeletal system disorder and the main symptom is bone mineral density reducing, which is an important cause of bone fractures and even a cause to threaten to the lives in elderly people.^[2]^ Approximately 200 million people with osteoporosis all over the world and 8.9 million osteoporosis fractures up to 2016.^[3]^ Vertebral fracture is the most common complication with osteoporosis, then following pain,^[4,5]^ which bring a lower quality of life and high costs for individual and the society. Unfortunately, there are no usual treatments for patients suffered from osteoporosis and only a small part of patients have access to rehabilitation programs according to the guidelines for osteoporosis.^[6]^

### 1.2 Description of the intervention

Mind-body exercise including taichi, dance, yoga, and Pilates, is regarded as a complementary therapy, which is beneficial for improving flexibility, controlling body balance, and reducing pain. Several clinical trials and meta-analysis studies have reported that exercise had positive effect on pain and quality of life in elderly people with osteoporosis.^[7–9]^

### 1.3 Why it is important to do this review

The high prevalence and heavy costs of osteoporotic-related fractures in aging people means prevention and management of this condition is important. Mind-body exercise on bone as a non-pharmacological intervention has been focused. A systematic review is required to carry in this area and summarize the evidence for clinical healthcare, policy makers, and all with an interest in this area. So in this study, a network meta-analysis will be carried out to assess the effect of mind-body exercise on osteoporosis in aging people, and will summarize a prioritization regimen.

## 2 Methods

### 2.1 Registration

This network meta-analysis has registered under PROSPERO (CRD42020165385), and will be reported following the Preferred Reporting Item for Systematic Reviews and Meta-Analysis Protocols (PRISMA-P) checklist.

### 2.2 Search strategy and data sources

**Figure 1:**
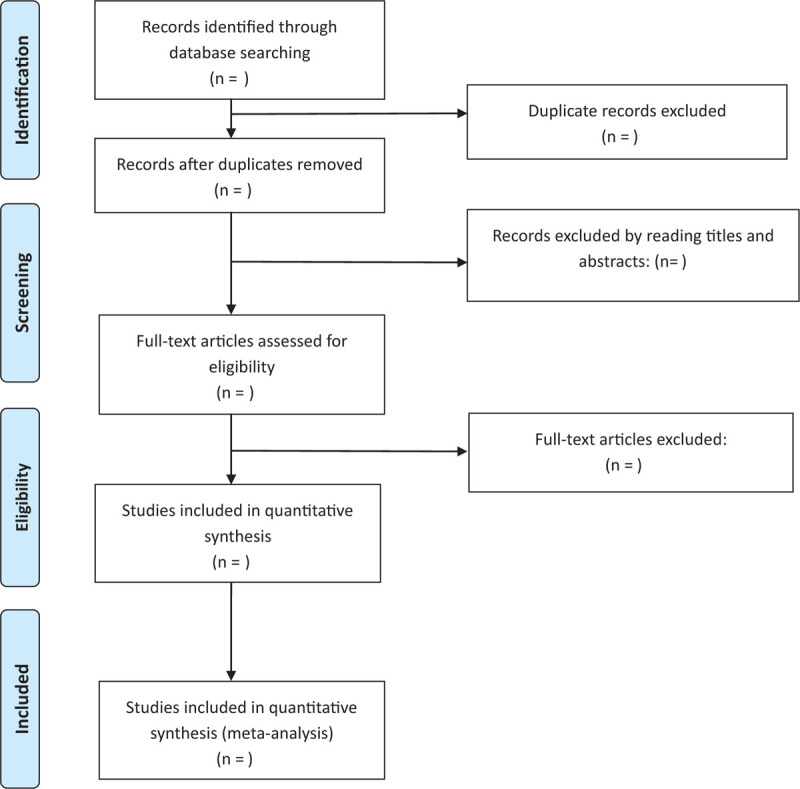
Literature screening process.

Web of Science, PubMed, Science Direct, Medline, Cochrane Library, China National Knowledge Infrastructure (CNKI), and Wanfang database will be searched from inception to January 2020. The searching strategies will combine Medical Subject Headings and free-text terms including osteoporosis, mind-body exercises, and clinical trials. (Table 1, Fig. 1).

**Table 1:**
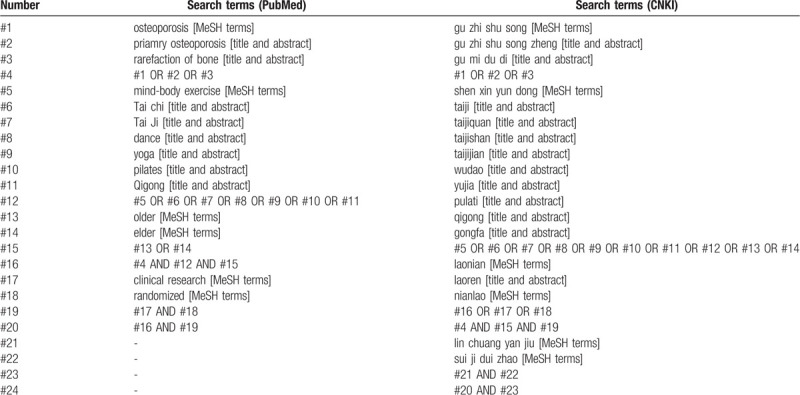
Search strategy.

### 2.3 Criteria for considering studies for this review

#### 2.3.1 Types of included studies

All controlled clinical trials including randomized controlled trial (RCT), parallel-group study, and retrospective controlled trial evaluating one or more following outcome: bone mineral density (BMD), bone mineral content (BMC), quality of life (QOL), balance, six-minute walking test (SMWT), and sit-to-stand test (SST). There will be no regardless of published language, blinding, and publication status of those included studies.

#### 2.3.2 Types of included participants

Included participants must be diagnosed older adults with osteoporosis in the observation group without respective of sex, ethnicity restrictions, nationality, or duration of disease.

#### 2.3.3 Types of interventions

Interventions in observation groups will be mind-body exercises including taichi (taichi quan, taichi shan, taichi jian), dance, yoga, and Pilates. Interventions in comparisons groups will be no regular exercise, or other exercises (i.e., jogging, walking).

#### 2.3.4 Types of outcome measures

The primary outcome will be global assessment for osteoporosis (i.e., BMD), and the secondary outcomes will be other indexes in connection with osteoporosis (i.e., QOL, BMC, SMWT, SST, balance).

### 2.4 Data extraction

Study ID, sample size, age of patients, interventions, outcomes, and duration of exercise will be extracted in a table.

### 2.5 Risk of bias and reporting quality of included trials

Two authors (ZYH and WZJ) will assess quality of reporting and methodology independently by using Cochrane Review Handbook. Seven domains including selection bias, performance bias, detection bias, attrition bias, reporting bias, and other bias would be judged with low, unclear, and high risk. Discussions should be carried out and a third author (WHJ) should give final decision when there were disagreements.

### 2.6 Statistical analysis

Review Manager 5.3 software (Cochrane Collaboration, UK) will be used for meta-analysis. Mean difference (MD) with 95% confidence interval (CI) will be used for analyzing continuous data. *I*^*2*^ statistics are calculated to the heterogeneity and to choose the effect model. Statistical heterogeneity will exist among included trials when *I*^*2*^ > 50% and *P* value <.1, so that a random-effects model would be selected; otherwise a fixed model will be chosen. Subgroup analysis would be carried out by different interventions or if the pooled results included clinical heterogeneity.

#### 2.6.1 Subgroups analysis

Subgroup analyses will be preformed based on different body parts of osteoporosis, age of participants, and sex. However, subgroup analysis will depend on enough data so it is hard to decide in advance.

#### 2.6.2 Sensitivity analysis

Sensitivity analysis will be carried out to determine data reliability according to sample size, performed geographical areas, missing data, and other relevant situations.

#### 2.6.3 Evidence network diagram

Dichotomous data will be transformed into the format shown in Table 2, and continuous data are in Table 3. The Stata 13.0 software page package (StataCorp, College Station, TX) will be used for analysis the evidence network diagram.

**Table 2:**
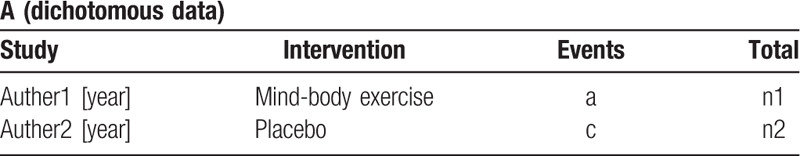
Data form for dichotomous data.

**Table 3:**
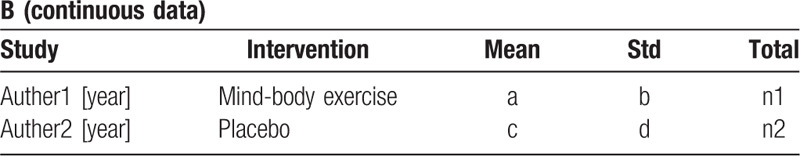
Data form for continuous data.

#### 2.6.4 Inconsistency examination

The consistency assumption will be assessed for the whole network by the design-by-treatment interaction model.^[10]^ Inconsistency data will be evaluated by triangular loops, which are closed loops and will be divided into different loops when there are >3 interventions. Inconsistency factor will be used for assessing (with 95% CIs^[11]^ and *z*-test) between direct and indirect estimates for every paired comparison.

#### 2.6.5 Ranking of effects

Based on design-by-treatment interaction, all interventions and their rank of effectiveness will be conducted by the surface under the cumulative ranking curve value.

#### 2.6.6 Publication bias

A funnel plot to assess the publication among the included trials will be used according to the publication bias might decrease the evidence intensity.^[12]^

### 2.7 Evidence quality grating

Evidence quality will be conducted by the Graduates Assessments, Development and Evaluation (GRADE) established by the World Health Organization with the results of high quality, medium quality, low quality, and very low quality.

### 2.8 Patients and public participation

This is a systematic review and network meta-analysis, so there will be not involve patient and public data collection.

### 2.9 Ethics and communication

Since this systematic review will do not involve raw data collection, no ethical review is required. The results from this study would provide some available evidence for mind-body exercise for older adults with osteoporosis. This systematic review will be published in a peer-reviewed journal.

## 3 Discussion

Mind-body exercise is a kind of regular exercise alone or in a team, which could promote both physical and psychological health.^[13,14]^ It is very suitable for older adults because of the gentle and slow movement, coordination of body and breathing, and easy learning and practicing.^[15–19]^ As non-drug interventions, mind-body exercises have been proven in favor of improving conditions of cardiovascular disorder,^[20]^ osteoporosis,^[21]^ pain and insomnia,^[22,23]^ and also some mental disorders.^[24,25]^

Older adult is a group who need more attention because not only their low quality of physiology health but also the inner loneliness. Osteoporosis is a common condition which limits the activity of movement, brings the feeling of pain, and lost the chance to communicate with others in older adults.^[26]^ Osteoporosis is associated with increased mortality and decreased health-related quality of life in older people, and a prediction shows that $25.43 will cost for osteoporosis related condition per year by 2050.^[27]^ Mind-body exercise seems to be a low-cost and effective intervention for older adults with osteoporosis from the results of the previous studies.^[28–30]^

However, no guidelines about mind-body exercise for older adults with osteoporosis have been developed. This systematic review will demonstrate evidence on efficacy and safety of various mind-body exercises for osteoporosis, and a possible regimen for mind-body exercises, which may be helpful for older adults with osteoporosis, clinical practitioners, guideline makers, and all who are interested in this field.

## 4 Conclusion

Some available evidence from this systematic review may be helpful for older adults with osteoporosis, clinical practitioners, guideline makers and all who are interested in this field.

## Author contributions

YHZ and ZJW did the equal contribution for this study, so they were the co-first authors for this study. YHZ and HXY were the guarantor of integrity of this entire study and wrote this manuscript. WQC and LM did study design, and date acquisition was by HJW and ZJW, and methodology analysis was by YHZ and HJW. Manuscript editing and review were by all the authors.
